# A Dangerous Operation

**Published:** 1886-09

**Authors:** 


### A DANGEROUS OPERATION.

In a paper read before the Texas State Medical Association by Prof. Edmond Souchon, of Tulane University, Dr. Souchon tells of an amusing occurrence that attended an operation in the Charity hospital clinic, as follows:

It was a case of abscess of the axilla, and as it was intended to make only one thrust of the knife, it was thought unnecessary to push the chloroform to a full anæsthesia; so the patient was only stupified a little, and the abscess lanced.

The Doctor says: "As the knife penetrated the flesh, the patient sprang to his feet in a wild, excited and unconscious manner, rushed at the case of instruments, which was near by, and had half opened a drawer and was about seizing an instrument, when one of the students bravely caught him. He very soon quieted down. The next day, in the ward, he told us that he had just come from a grinding on a sugar plantation, where a number of Chinese were employed; that he was always quarreling with them, and that as he was going under chloroform, the students around him became gradually transformed, in his mind, into Chinese; and that as the knife penetrated him, though he felt no pain, he felt satisfied it was one of the Chinamen who had stabbed him. Hence the rush he made to what he thought was his armory, for a weapon to defend himself."

